# Normal Ovariotomy, Case, Recovery

**Published:** 1875-02

**Authors:** T. T. Sabine


CASE OF NORMAL OVARIOTOMY; RECOVERY; ST. LUKE'S HOSPITAL, NEW YORK—*By T. T. Sabine, M.D. Reported by Geo. Hart, M.D., House Surgeon.*—A. B., aged twenty-five; single; United States; admitted June 17, 1874. Patient was perfectly well up to eight years ago, at which time, while menstruating, she took a cold bath, which was followed by cessation of the menstrual flow, and a very severe attack of neuralgic pain in the left iliac fossa and left limb, lasting several weeks, and resembling, only in a much milder form, the pain which she has since suffered. The treatment was by leeches, wet cups, etc.

After this the catamenia became extremely painful, and dysmenorrhœa was constant for four years. At this time (four years ago) the dysmenorrhœa became more intense, and was accompanied by severe neuralgic pain, limited to the region of the left ovary. Patient remained in this condition until eighteen months ago, when there was a sudden increase in the severity of the attacks, probably due to frequent exposures to wet and cold. The catamenia ceased for three months, and corresponding to the menstrual periods there were attacks of intense ovarian pain, lasting about ten days. The treatment at this time was by leeches and ice locally, and the internal administration of morphine.

In the spring of 1873 patient suffered attacks of increased severity, the pain being so great as to produce convulsions, and was only relieved by chloroform.

In September, 1873, she was operated on at the Woman's Hospital in this city, for dysmenorrhœa, but without relief. Subsequently an operation was performed for vaginismus, with a similar result.

During the past year patient has hardly ever been entirely free from pain, and has been unable to bear the weight of the body on the left limb, on account of the severe pain which it excited in the region of the left ovary.

The medical treatment of the paroxysms has been the free administration of morphine. In 1872 morphine was given in doses of half a grain to a grain and a half, repeated as required. As the attacks increased in violence, morphine failed to give relief, and chloroform or ether was given by inhalation.

On admission, patient is in fair general condition. She can get about the ward on crutches, but cannot bear the weight of the body on the left limb, which is fixed and lies across the right as in morbus coxarius.

Physical examination reveals intense vaginismus, and exquisite tenderness in the region of the left ovary. By conjoined manipulation the ovaries can be easily felt, and are of normal size. After this examination, as after every previous one, patient suffered great pain for two or three days.

July 4.—Patient was seized with a paroxysm of pain referred to the left ovary, so great as to cause convulsive action of all the muscles of the body, and which sol. morph. sulph. Magendie, gtt. xl, hypodermically, and repeated in half an hour, failing to control, chloroform was administered in quantities sufficient to relieve the pain. This was continued for six hours, when the suffering abated. Moderate relief was obtained for twenty hours, the pain being controlled by morphine, but at the end of that time she was again seized with a paroxysm which lasted, with intermissions, for three days, at times requiring the administration of chloroform in addition to large doses of morphine. Great exhaustion followed each severe paroxysm, and the patient was considerably prostrated by the prolonged attack.

July 19.—Patient again suffered from an attack of unusual severity, lasting twelve hours, which was only relieved by chloroform; and on July 26 another attack, lasting fifteen hours, was relieved by the same means. Both were followed by extreme exhaustion and weakness.

July 31.—A consultation was held, and removal of the ovary decided upon. Patient is failing in health and strength, and is very anxious for relief. General condition fair. Urine normal.

August 8.—Operation by Dr. T. T. Sabine, Drs. H. B. Sands and J. L. Little assisting. Temperature of the operating room 80°. Patient being etherized, an incision was made in the median line from a point five inches above the pubes downward to within an inch of the latter point. The fascia covering the rectus muscle was divided, the incision carried along the inner edge of this muscle down to the peritonæum, and after all hemorrhage had ceased it was carefully divided upon a director.

The hand was immediately passed through the wound to the fundus of the uterus, and thence along the broad ligament to the left ovary, which was found free from adhesions, and was readily seized and brought out at the wound. An eye-probe, threaded with two carbolized cat-gut ligatures, was passed through the broad ligament just below the ovary, the ligatures firmly tied on either side, the ovary cut off with scissors, and the pedicle, which was perfectly dry, replaced in the abdominal cavity, into which only a few drops of blood had escaped from the wound.

Four silver sutures, passing through the abdominal parietes and peritonæum on either side, were introduced and the wound tightly closed. The integument was brought together between the wires by four silk sutures, and straps of adhesive plaster, with compress and bandage, applied.

The ovary was of natural size, and on section the stroma and capsule appeared normal. The very unusual opportunity was afforded of examining a corpus luteum, the exact age of which was known immediately after the removal of the ovary from the living body. The patient had menstruated just three weeks prior to the operation, and the corpus luteum, examined by Dr. J. C. Dalton, answered perfectly the description of the one represented in his work on "Human Physiology," page 566, of the fourth edition, excepting that it projected much more prominently from the surface of the ovary.

Evening.—Patient has rallied well from the ether. Complained of diffuse pain in the abdomen, which was controlled by morphine administered hypodermically. No nausea.

August 9.—Last night patient was very restless and suffered much from abdominal pain, but is more comfortable this morning. Morphine has been administered hypodermically as required.

Menstrual flow appeared this morning, accompanied by the usual dysmenorrhœal pain, but none in the region of the ovary.

August 10.—Patient passed quite a comfortable night, sleeping quietly for four hours. Toward morning more restless. Menstrual flow has ceased. Morphine administered by the mouth, still in considerable quantities. Very little constitutional disturbance.

August 11.—Slept pretty well last night, but when awake was very restless, and suffered considerable pain in the abdomen. This morning is much better.

August 13.—Doing remarkably well. Constitutional disturbance very slight.

August 15.—All the silk and the upper silver sutures removed. Union perfect throughout the entire length of the wound. Is very comfortable, and almost entirely free from pain.

August 19.—Suffered considerably last night from rectal tenesmus, which was in part controlled by suppositories. This morning an enema of castor oil was administered, followed by a free evacuation from the bowels, which gave great relief.

August 30.—Patient very comfortable, and up every day.

September 7.—Patient has been out on the lawn two or three times during the last week, and is entirely free from pain in the region of the ovary.

General health very much improved. The left limb, which at the time of admission was semi-flexed, so that the foot could not be brought to the floor, is now perfectly straight, and easily sustains the weight of the body in walking, although it is still weak from disuse.

September 12.—Discharged, cured.

September 29.—Patient seen to-day. Can walk easily, and without limping, for a considerable distance. Has menstruated since leaving the hospital, with entire absence of ovarian or dysmenorrhœal pain.

